# Analgesic efficacy of erector spinae plane block for managing pain in arthroscopic shoulder surgery: a systemic review and meta-analysis

**DOI:** 10.3389/fmed.2025.1702898

**Published:** 2025-12-12

**Authors:** Ying Wang, Qiuya Yang, Wen Jie, Yan Liu, Fenglin Jiang

**Affiliations:** 1 Department of Emergency, Clinical Medical College and Affiliated Hospital of Chengdu University, Chengdu, Sichuan, China; 2 Hospital Infection Management Department, Clinical Medical College and Affiliated Hospital of Chengdu University, Chengdu, Sichuan, China; 3 Department of Nursing, Pengzhou Peoples’s Hospital, Pengzhou, Sichuan, China

**Keywords:** erector spinae plane block, analgesia efficacy, arthroscopic shoulder surgery, pain management, meta-analysis

## Abstract

**Background:**

Whether the erector spinae plane block (ESPB) truly relieves pain after arthroscopic shoulder surgery (ASS) is still unsettled. We therefore examined whether ESPB sharpens post-operative pain control in these patients.

**Methods:**

We systematically searched the Cochrane Library, PubMed, Embase, and Web of Science for randomized controlled trials (RCTs) comparing ESPB with any comparator (no block, sham block, or alternative regional block) in patients undergoing ASS. The primary outcome was cumulative opioid consumption within the first 24 h postoperatively. Secondary outcomes included pain scores at rest and during movement, incidence of postoperative nausea and vomiting (PONV), time to first rescue analgesic request, and patient-reported satisfaction with analgesia.

**Results:**

Six RCTs comprised of 365 patients met inclusion criteria. ESPB did not reduce 24-h opioid consumption versus control (SMD −1.11; 95% CI −2.55 to 0.33; *p* = 0.13, *I*^2^ = 96%). Pain scores were lower with ESPB at 2 h (SMD −0.83; 95% CI −1.30 to −0.37; *p* = 0.0005, *I*^2^ = 35%) and 48 h (SMD −0.64; 95% CI −1.08 to −0.20; *p* = 0.004, *I*^2^ = 95%), but not at 4 h. Furthermore, time to first rescue analgesic was prolonged by ESPB (SMD 4.04; 95% CI 0.77–7.31; *p* = 0.02, *I*^2^ = 99%). However, ESPB did not reduce the rest and movement pain scores at 2 h (SMD −0.87; 95% CI −2.98 to 1.24; *p* = 0.42; *I*^2^ = 97%; SMD −0.98; 95% CI −3.00 to 1.04; *p* = 0.34; *I*^2^ = 97%) and 4 h (SMD −0.43; 95% CI −2.31 to 1.46; *p* = 0.66; *I*^2^ = 97%; SMD −0.89; 95% CI −2.57 to 0.80; *p* = 0.30; *I*^2^ = 96%), respectively. PONV and other adverse events were comparable. Subgroup analysis of single-injection ESPB also showed no opioid-sparing effect (SMD −1.46; 95% CI −3.21 to 0.30; *p* = 0.10, *I*^2^ = 97%). Patient-reported satisfaction revealed no significant difference between ESPB and control group.

**Conclusion:**

The ESPB fails to reduce 24-h opioid consumption, pain scores at rest and movement at early stage, and the incidence of PONV. Nevertheless, it prolonged the time to first rescue analgesic without elevating the risk of adverse events.

**Systematic review registration:**

PROSPERO, registration number CRD 42023395027, https://www.crd.york.ac.uk/PROSPERO/view/CRD42023395027.

## 1 Introduction

Modern arthroscopic techniques have converted most shoulder operations into true day-case procedures (1), yet 54% of patients still experience moderate-to-severe pain afterwards, a burden that can derail early mobilization and precipitate unplanned readmission (1, 2). Effective, early analgesia is therefore the linchpin of rapid rehabilitation and shortened length of stay. Regional blockade has become the cornerstone of any multimodal regimen for these patients; the interscalene brachial-plexus block long held pride of place (3), but its well-documented collateral effects have fueled an active search for safer alternatives (3).

Recent work shows that a suprascapular-nerve block (SSNB) can deliver reliable analgesia while leaving the phrenic nerve untouched (4). Yet the lingering side-effects of opioids, Interscalene brachial plexus block (ISB) or even SSNB continue to complicate effective pain relief (3). Because sound rehabilitation and the functional gains that follow hinge on keeping pain in check after arthroscopic shoulder surgery (ASS), a truly efficient analgesic strategy is indispensable for improving outcomes. Introduced by Forero et al. in 2016 for refractory thoracic pain (5), the erector-spinae plane block (ESPB) has since migrated into the peri-operative arena. When injected at the correct spinal level the technique is technically undemanding and can bathe dermatomes from T1 to L3 (3, 6–11). Forero’s group subsequently showed that a high-thoracic ESPB also tames chronic shoulder pain (4), and Taysser et al. reported superior analgesia after ASS compared with intra-articular bupivacaine (12). Bahadir et al. likewise found ESPB outperforming sham injection (13), yet a head-to-head trial against the ISB favored the latter (3). These conflicting signals leave the block’s true worth in ASS uncertain, prompting a fresh wave of randomized trials that now invite quantitative synthesis.

This systematic review and meta-analysis aimed to synthesize evidence from randomized controlled trials (RCTs) to evaluate the analgesic efficacy and safety of ultrasound-guided ESPB in patients undergoing ASS. By addressing these research questions, we seek to provide clinicians with evidence-based recommendations for integrating ESPB into multimodal analgesic regimens, ultimately optimizing postoperative outcomes in this patient population.

## 2 Methods

The present systematic review and meta-analysis was conducted in strict accordance with the PRISMA 2020 statement and the AMSTAR-2 checklist to ensure transparent reporting and methodological rigor (13). An a-priori protocol detailing the review question, eligibility criteria, search strategy, data-extraction procedures, and statistical analyses was prepared and registered with the International Prospective Register of Systematic Reviews (ROSPERO, registration number CRD 42023395027) before any study selection began. Throughout the project we followed this protocol without deviation, used only anonymized summary data already available in the public domain, and collected no new information from living individuals. Consequently, the work was deemed secondary research and was formally exempted from institutional review-board approval and from the requirement for informed consent.

### 2.1 Search strategy

We systematically interrogated the Cochrane Library, PubMed, Embase and Web of Science from inception to March 2023, with no language or date restrictions initially imposed, coupling MeSH headings with free-text strings and imposing neither language nor date limits. The search was built around the concepts of “arthroscopic shoulder surgery” (shoulder arthroscopy, total shoulder arthroplasty, shoulder surgery) and “erector spinae plane block” (erector spinae muscle), using Boolean operators and truncation to capture every relevant permutation (full line-by-line strategy supplied in Supplementary Digital Content 1). To pre-empt duplication and benchmark our methods against earlier syntheses, we also ran parallel retrieval for existing systematic reviews. Grey literature was hunted manually through conference proceedings, trial registries and thesis repositories, while the reference lists of every eligible article were snowballed to ensnare any overlooked trials.

### 2.2 Study selection criteria

Two reviewers working independently screened every record against the prespecified criteria, first on title and abstract and then on the full text. Each made the final inclusion or exclusion decision without knowledge of the other’s choice; disagreements were settled by open discussion until consensus was reached. We retained randomized controlled trials that enrolled adults having arthroscopic shoulder surgery includes arthroscopic rotator cuff repair, labral repair, and arthroscopic-assisted total shoulder arthroplasty under general anesthesia, compared any form of ESPB (single-shot, a single intraoperative or preoperative injection of local anesthetic; or continues ESPB, involving an indwelling catheter for continuous local anesthetic infusion) with an alternative regional technique, sham injection, or no block, and quantified post-operative opioid consumption in both study arms. Case reports, observational studies, conference abstracts, letters and trials still recruiting were all excluded.

### 2.3 Data extraction

Two reviewers working independently extracted data from every included study with a standardized, pre-piloted form. Whenever their entries diverged, a third reviewer was consulted to reach consensus. The form captured bibliographic details (authors, year, country, sample size, sex distribution, ASA class), design elements (randomization method, control and intervention descriptions), pain-related endpoints (cumulative opioid dose in the first 24 h, pain scores at rest and on movement, time to first rescue analgesic), and additional metrics (postoperative complications, patient satisfaction scores). Cumulative opioid consumption at 24 h postoperatively was predefined as the primary outcome; all other variables were classified as secondary.

For the quantitative synthesis, means and standard deviation (SD) were retrieved for continuous or ordinal endpoints such as opioid use or pain intensity, while event counts were collected for binary outcomes like postoperative nausea and vomiting (PONV). When authors reported medians, interquartile ranges, or full ranges, we converted these statistics to means and SD with the approximation algorithms proposed by Hozo and colleagues or those outlined in the Cochrane Handbook (14, 15). All opioid doses were translated into oral morphine equivalents (OME) with a validated conversion table (16). Pain at rest or movement was measured with either the visual analogue scale (VAS) or the numeric rating scale (NRS); because the two instruments behave interchangeably (17), each score was linearly transformed to a common 0–10 metric. Assessments were scheduled at fixed intervals: on arrival in the post-anesthesia care unit and at 1, 2, 4, 8, 24, and 48 h after surgery.

### 2.4 Assessment of methodological quality

Two reviewers independently appraised the methodological rigor of every trial. For the randomized controlled trials, we applied the Cochrane risk-of-bias tool (18), which scrutinizes seven domains: random-sequence generation, allocation concealment, blinding of participants and personnel, blinding of outcome assessment, completeness of outcome data, selective outcome reporting, and any additional threats to validity. Each domain was classified as low, high, or unclear risk, informing an overall judgment of the study’s susceptibility to selection, performance, detection, attrition, or reporting bias. The certainty of evidence for each pooled outcome was graded with the Grading of Recommendations, Assessment, Development and Evaluations (GRADE) approach, considering study limitations, inconsistency, indirectness, imprecision, and publication bias (19).

### 2.5 Statistical analysis

Continuous endpoints included 24-h opioid consumption, pain intensity, time-to-first analgesic request, and patient satisfaction scores were pooled as standardized mean difference (SMD) with 95% confidence interval (CI) under a random-effects inverse-variance model, chosen because the metric is clinically interpretable across heterogeneous scales (20). Dichotomous events, including PONV, respiratory depression, pruritus, or local-anesthetic toxicity were summarized as risk ratios (RR) with 95% CI via the Mantel–Haenszel method (20). Heterogeneity was quantified with Cochran’s Q and *I*^2^; *I*^2^ > 50% flagged substantial inconsistency (20). A random-effects framework was retained throughout to accommodate variation in block technique among operators. Subgroup comparisons were restricted to the primary outcome, OME use within 24 h, among trials that delivered a single-injection ESPB. Robustness was explored by leave-one-out sensitivity analysis for the same endpoint. Funnel-plot asymmetry tests (21) evaluated small-study effects in the primary outcomes. Due to the small number of included studies, Egger’s regression and Begg’s rank correlation tests were not performed, as these tests have lower power for less than 10. Two-tailed *p* < 0.05 defined statistical significance. All computations were performed in Review Manager (RevMan) version 5.3.

## 3 Results

### 3.1 Identification of studies

**Figure 1:**
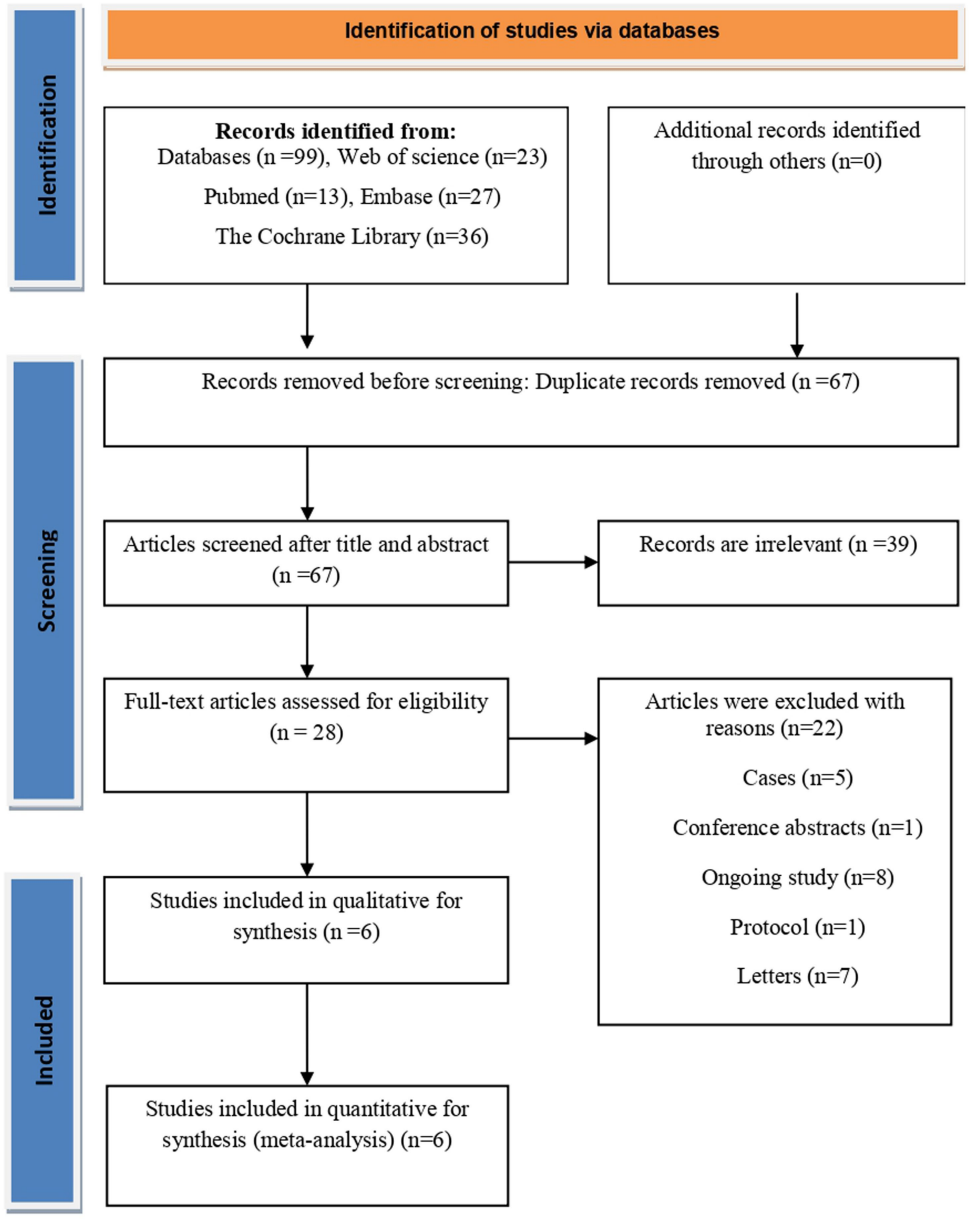
The PRISMA flow diagram of included study of this network-meta-analysis.

The PRISMA flow-chart (Figure 1) illustrates the step-by-step study selection process. Ninety-nine suitable articles, journals, and abstracts were found in the initial literature review. After thoroughly analyzing the papers and removing any duplicates, six of these studies (3, 11, 12, 22–24) were retained in the current systematic review.

Table 1 summarizes the key attributes of the six original research articles that met inclusion criteria (3, 11, 12, 22–24), all of which were registered on the Clinical Trials website. Across the six trials, 365 participants, 166 allocated to ESPB and 199 to control group contributed data to the present analysis. One trial (12) compared ESPB with shame block with 30 mL 0.9% saline at T2, and five trials with other blocks (3, 11, 22–24) (IAI with 20 mL 0.25% bupivacaine, ISB with 30 mL 0.25% bupivacaine, PAI with 30 mL 0.25% bupivacaine, and SSNB with 0.25% 10 mL, respectively). Two studies (22, 24) compared ESPB with continue-ISB (CISB) under different local anesthetics and found that ISB was superior to ESPB (22), however, CESPB could provide a phrenic nerve-sparing alternative analgesia efficacy to CISB (22).

**Table 1:** Overview of demographic details of ESPB in arthroscopic shoulder surgery.

Study	Regime	No. of patients	Age	Gender (M/F)	ASA	Anesthesia	Surgery	Intervention	Control	Sensory test	Multimodal analgesia protocol	Pain scale (rest and movement)	Outcomes	Findings	Registry website
Taysser et al. (11)	Egypt	30 vs. 30	33.3 ± 9.08 vs. 35.7 ± 9.52	17/13 vs. 11/19	I-II	GA	ASS	ESPB at T2 with 20 mL 0.25% bupivacaine + sham IAI with 20 mL saline	IAI with 20 mL 0.25% bupivacaine + sham ESBP with mL saline	NA	NA	VAS	[1, 2, 3, 5]	ESPB superior to IAI	ClinicalTrials.gov (NCT04483323)
Bahadir et al. (12)	Turkey	30 vs. 30	47.6 ± 13.01 vs. 49 ± 10.26	10/20 vs. 16/14	I-II	GA	ASS	ESPB at T2 with 30 mL 0.25% bupivacaine	Sham block at T2 with 30 mL 0.9% saline	Cold test	① 400 mg ibuprofen and 100 mg tramadol ② PCA	VAS	[1, 2, 3]	ESPB superior to sham block	ClinicalTrials.gov (NCT04081948)
Furkan et al. (22)	Turkey	30 vs. 30	47.03 ± 13.3 vs. 45.07 ± 14.72	15/15 vs. 17/13	I-II	GA	ASS	ESPB at T2 with 30 mL 0.25% bupivacaine	ISB at T2 with 30 mL 0.25% bupivacaine	Cold test	① 400 mg ibuprofen and 100 mg tramadol ② PCA	VAS (both)	[1, 2, 3, 4, 5]	ISB provided more effective analgesia than ESPB	ClinicalTrials.gov (NCT04083287)
Shanthanna et al. (23)	Canada	31 vs. 31	44.8 ± 15.8 vs. 43.8 ± 15.8	26/5 vs. 18/13	I-III	GA	ASS	ESPB at T2 with 30 mL bupivacaine 0.25% with 5 μg.ml-1 adrenaline +PAI	PAI with 30 mL 0.25% bupivacaine with 5 μg.ml-1 adrenaline +ESPB	Cold test	① 650 mg acetaminophen.	NRS (both)	[1, 2, 3, 4, 5, 6]	ESPB was not superior to PAI	ClinicalTrials.gov (NCT03691922).
Lisa et al. (24)	USA	12 vs. 14	72.3 ± 6.5 vs. 70.4 ± 12.4	7/5 vs. 10/4	I-III	GA	TSA	Continue ESPB at T1/T2 with 0.5% ropivacaine	ISB with 0.5% ropivacaine	NA	acetaminophen, gabapentin, oxycodone, and hydromorphone	NRS	[1, 3, 4, 5, 6]	CESPB provided alternative analgesia efficacy	ClinicalTrials.gov (NCT03807505)
Naglaa et al. (3)	Egypt	33 vs. 32 vs. 32	45.9 ± 6.7 vs. 47.5 ± 7.9	25/7 vs. 22/10	I-II	GA	ASS	ESPB at T2 with 30 mL of the LA bupivacaine 0.25% and epinephrine 5 μg/mL	① SSNB with 0.25% 10 mL bupivacaine with epinephrine 5 μg/mL; ② no block	Cold test	acetaminophen (1 g PO)	NRS (both)	[1, 2, 3, 5]	SSNB not inferior to ESPB	ClinicalTrials.gov (NCT04669639)

ASS, Arthroscopic Shoulder Surgery; ESPB, erector spinae plane block; SSNB, Suprascapular nerve block; NRS, Numeric Rating Scale; IAI, Intraarticular injection; VAS, Visual analogue scale; PCA, patient control analgesia; ISB, Interscalene brachial plexus block; PAI, Peri-articular injection; TSA, Total shoulder arthroplasty; ASA, American society of Anesthesiologist; CESPB, Continue erector spinae plane block. Outcomes: [1] Pain scores at different points (NRS, VAS). [2] Postoperative opioids consumption (morphine, fentanyl use). [3] Rescue analgesics. [4] Opioids related complications. [5] Postoperative complications (postoperative nausea, itching, vomiting, and so on). [6] Patient satisfaction with analgesia.

Four studies investigated the postoperative opioid consumption at 24 h (3, 12, 22, 24). Three studies (11, 12, 24) investigated the pain scores (where there is no distinction between resting and movement pain), two studies looked at rest and movement at different points (3, 22) (NRS/VAS scores). Two studies found intraoperative fentanyl consumption (3, 12), two looked at the prevalence of PONV (3, 23), and two looked at nausea (12, 22) and vomiting (12, 22). Respiratory depression was investigated in three studies (3, 12, 23), postoperative itching was investigated in three studies. LAST linked to regional analgesia techniques was discovered in two studies (23, 24), the first use of rescue analgesics was recorded in three studies (3, 11, 23), patients’ satisfaction with analgesia methods demonstrated in two studies (23, 24). Table 2 collates the secondary outcomes and the GRADE certainty ratings. Across the six RCTs (365 participants) the Cochrane RoB-2 assessment showed low risk for randomization and incomplete outcome data in every trial, whereas allocation concealment remained unclear in four studies and blinding of participants/personnel was impossible (high risk) in three; detection bias was rated unclear in four trials, although the objective nature of the primary opioid-consumption outcome mitigates concern. No selective reporting or additional biases were detected. Overall, two studies were classified as low risk, three as “some concerns,” and one as high risk; yet leave-one-out sensitivity analyses confirmed that excluding the high-risk or any unclear-risk trial did not alter the significance or direction of the pooled estimates, indicating that the review’s conclusions are robust to the identified methodological limitations.

**Table 2:** Summary of the secondary outcomes and quality of evidence using GRADE.

Outcomes	Included studies	Participants	Mean difference (95% CI)	I^2^ (%)	*p*-value	Quality of evidence (GRADE)	References
Pain scores at different points (VAS/NRS)							
Pain scores at PACU	2	86	0.28 (− 0.94 to 1.50)	84	0.66	⊕ ⊕ ⊕⊕	(11, 24)
Pain scores at 1 h postoperatively	2	120	−2.24 (− 5.48 to 1.00)	98	0.18	⊕ ⊕ ⊕⊕	(11, 12)
Pain scores at 2 h postoperatively	2	120	− 0.83 (− 1.30 to − 0.37)	35	0.0005	⊕ ⊕ ⊕⊕	(11, 12)
Pain scores at 4 h postoperatively	2	120	1.74 (4.18 to − 7.65)	99	0.56	⊕ ⊕ ⊕⊕	(11, 12)
Pain scores at8h postoperatively	2	120	−2.45 (− 5.34 to 0.44)	99	0.56	⊕ ⊕ ⊕⊕	(11, 12)
Pain scores at 24 h postoperatively	3	146	−1.37 (− 3.30 to 0.57)	96	0.17	⊕ ⊕ ⊕⊕	(11, 12, 24)
Pain scores at 48 h postoperatively	2	86	− 0.64 (− 1.08 to − 0.20)	95	0.03	⊕ ⊕ ⊕⊕	(12, 24)
Pain score at rest at 2 h postoperatively	2	190	−0.87 (− 2.98 to1.24)	97	0.42	⊕ ⊕ ⊕⊕	(3, 22)
Pain score at rest at 4 h postoperatively	2	190	−0.43 (− 2.31 to1.46)	97	0.66	⊕ ⊕ ⊕⊕	(3, 22)
Pain score at movement at 2 h postoperatively	2	190	−0.98 (− 3.00 to1.04)	97	0.34	⊕ ⊕ ⊕⊕	(3, 22)
Pain score at movement at 4 h postoperatively	2	190	−0.89 (− 2.57 to 0.80)	96	0.30	⊕ ⊕ ⊕⊕	(3, 22)
Postoperative complications							
Nausea	2	120	−0.89 (− 2.57 to 0.80)	89	0.89	⊕ ⊕ ⊕⊕	(12, 22)
Vomiting	2	120	1.01(0.14–2.51)	78	0.99	⊕ ⊕ ⊕⊕	(12, 22)
Respiratory depression	3	252	1.00(0.40–2.51)	–	1.00	⊕ ⊕ ⊕⊕	(3, 12, 22)
Itching	3	183	1.14(0.16–8.22)	65	0.90	⊕ ⊕ ⊕⊕	(12, 22, 23)
Local anesthetics poisoning	2	88	7.0(0.38–130.10)	–	0.19	⊕ ⊕ ⊕⊕	(23, 24)
PONV	2	192	0.55(0.23–1.35)	36	0.19	⊕ ⊕ ⊕⊕	(3, 23)
The first time of rescue analgesics	3	250	4.04(0.77–7.31)	99	0.02	⊕ ⊕ ⊕⊕	(3, 11, 23)

NRS, Numeric Rating Scale; VAS, Visual analogue scale; PACU, Post-anesthesia care unit; PONV, Postoperative nausea and vomiting; GRADE, Grading of Recommendations, Assessment, Development and Evaluations.

**Figure 2:**
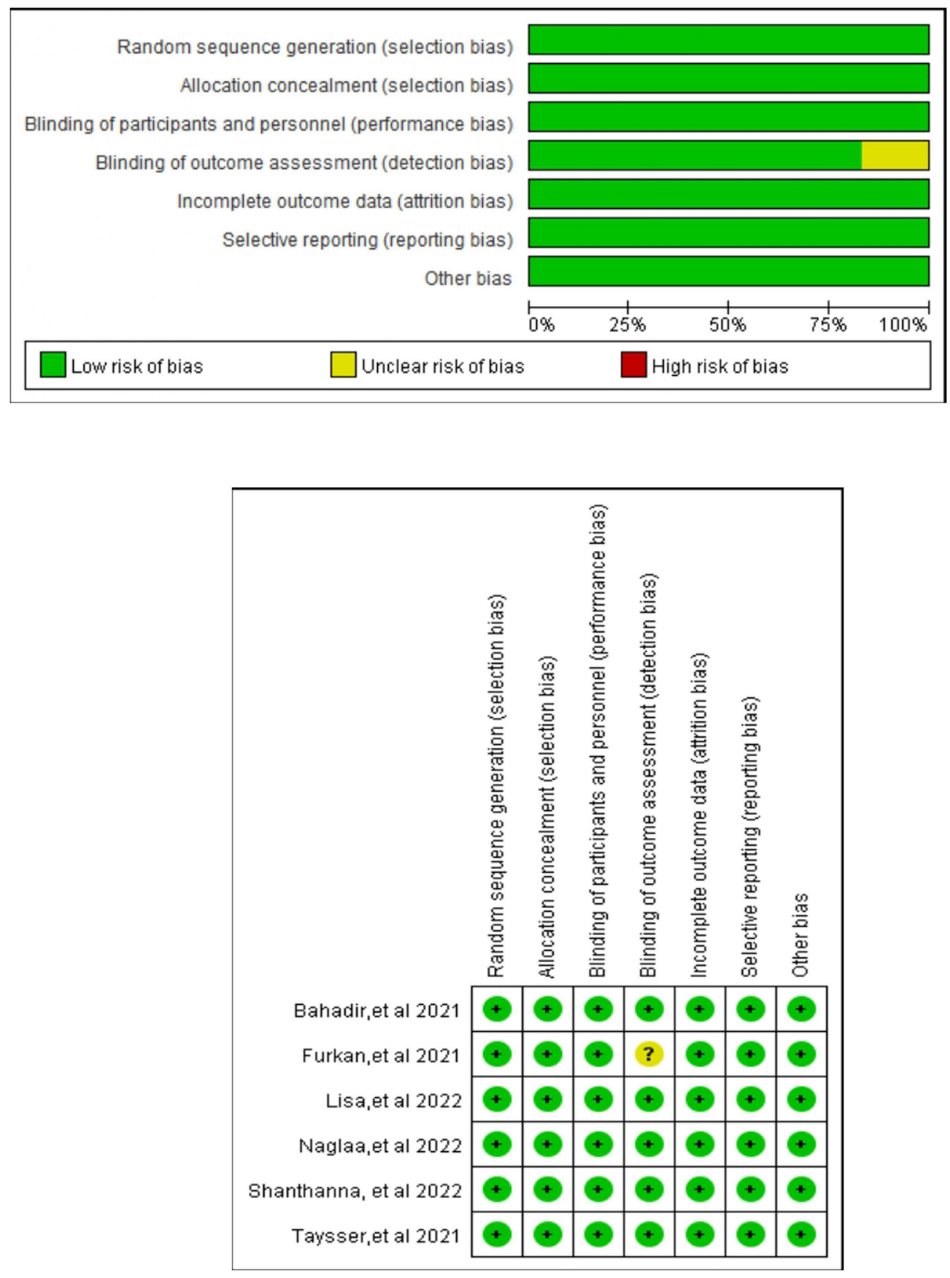
Risk of bias graph and summary: review authors’ judgments about each risk of bias item presented as percentages across all included studies. Green, red, and yellow circles indicate low, high, and unclear risks of bias, respectively (for interpretation of the references to color in this figure legend, the reader is referred to the web version of this article).

Figure 2 displays the Cochrane risk-of-bias judgments. It is a two-part graphic that color-codes the RoB-2 judgments for the six trials: the left bar chart shows, for each bias domain, the percentage of studies rated green (low risk), yellow (unclear) or red (high risk)—revealing unanimous low risk for randomization and incomplete data, two-thirds unclear for allocation concealment and assessor blinding, and half high risk for participant/personnel blinding—while the right matrix displays the same traffic-light symbols trial-by-trial, allowing readers to pinpoint which individual studies contribute the dominant concerns. Across the six trials, unclear allocation concealment and lack of participant blinding were the dominant methodological concerns. All studies had been prospectively registered at ClinicalTrials.gov (3, 11, 12, 22–24).

### 3.2 Opioid consumption in the first 24 h after surgery

**Figure 3:**
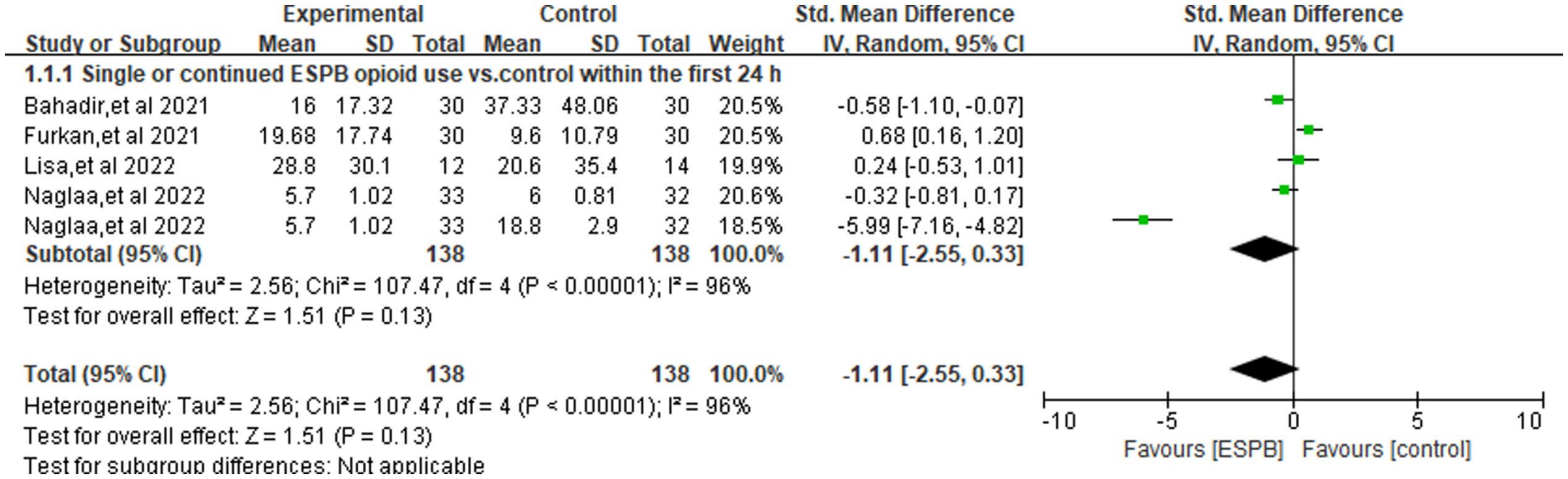
Forest plot comparing single or continued ESPB opioid use (orals morphine milligram equivalents) versus control within the first 24 h following surgery.

**Figure 4:**
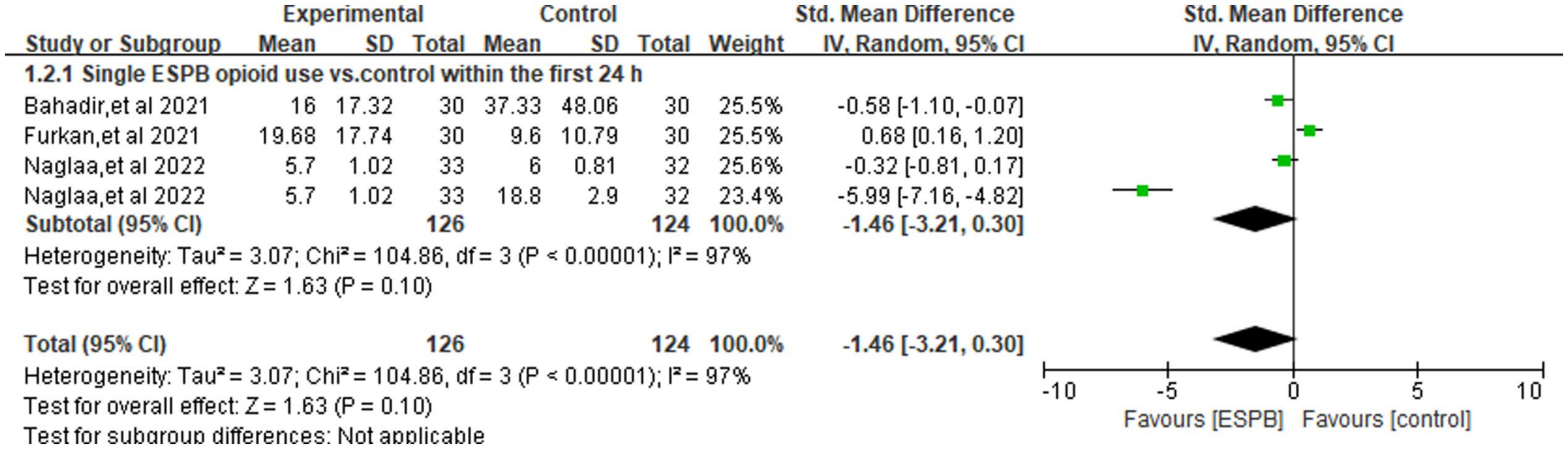
Forest plot comparing single ESPB opioid use (orals morphine milligram equivalents) versus control within the first 24 h following surgery.

Figure 3 illustrates the pooled comparison of 24-h opioid consumption between ESPB (single-shot or continuous) and control groups across four trials (3, 12, 22, 24), revealed there is no significant difference (SMD -1.11; 95% CI −2.55 to 0.33; =0.13, *I*^2^ = 96%). In a subgroup analysis limited to studies using a single-injection ESPB compared with control group (Figure 4), the reduction in opioid consumption was more pronounced but remained non-significant (SMD −1.46; 95% CI −3.21 to 0.30; *p* = 0.10, *I*^2^ = 97%). The funnel plot showed a little asymmetry, as shown in Supplementary Figure 3. Leave-one-out sensitivity analyses confirmed that the pooled estimate for 24-h morphine consumption remained directionally consistent and statistically non-significant after the sequential removal of any single trial, indicating that no individual study exerted undue leverage on the overall result, as shown in Supplementary Table 1.

### 3.3 Pain scores at different points postoperatively

**Figure 5:**
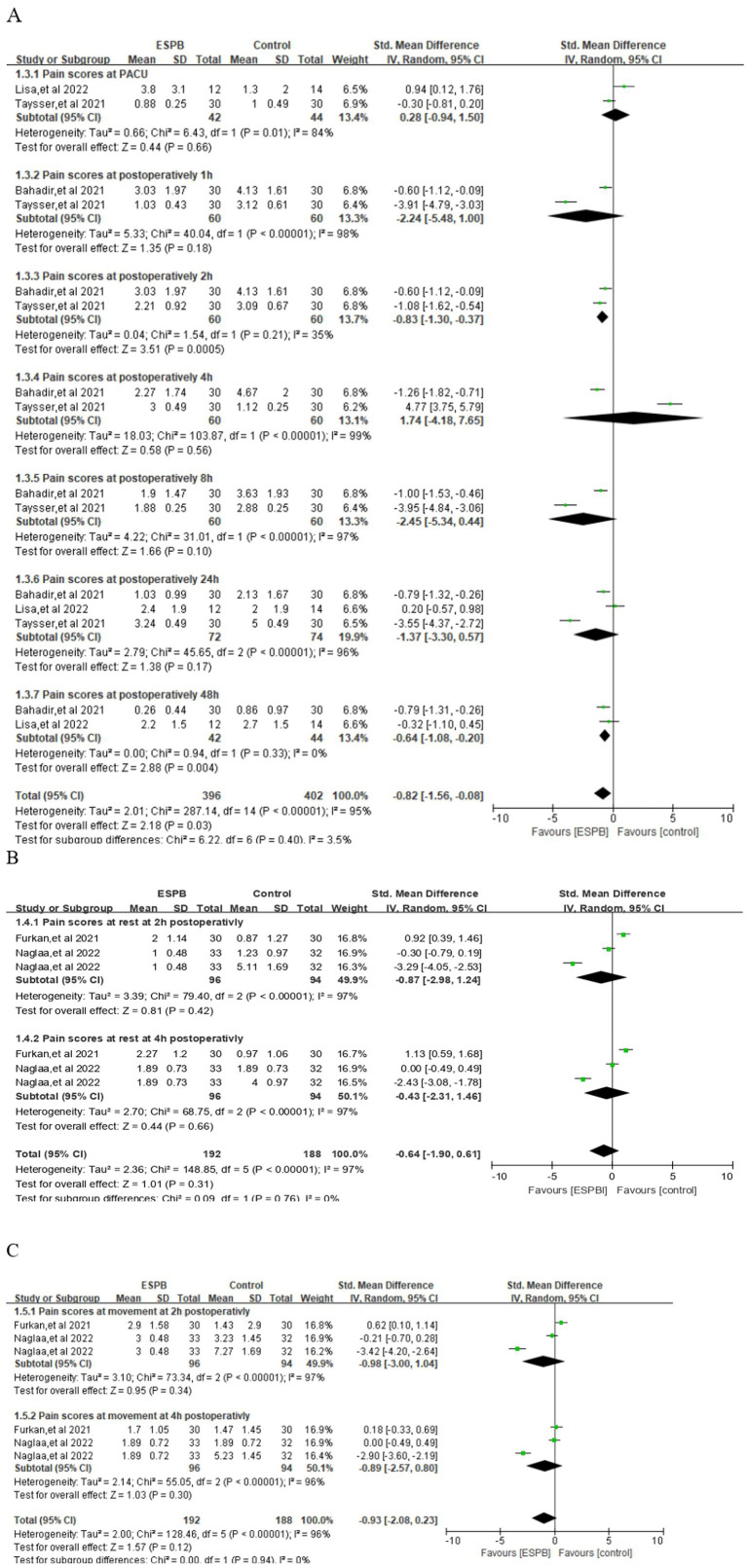
**(A)** Forest plots of the pain score at different point postoperatively. **(B)** Pain scores at rest at different point. **(C)** Pain scores at movement at different point.

ESPB produced a statistically and clinically meaningful reduction in pain at 2 h (SMD −0.83; 95% CI −1.30 to −0.37; *p* = 0.0005; *I*^2^ = 35%) and 48 h (SMD −0.64; 95% CI −1.08 to −0.20; *p* = 0.03; *I*^2^ = 95%) when compared with control (Figure 5A). In contrast, no reliable separation between groups was detected in the PACU (SMD 0.28; 95% CI −0.94 to 1.50; *p* = 0.66; *I*^2^ = 84%) or at 1 h (SMD −2.24; 95% CI −5.48 to 1.00; *p* = 0.18; *I*^2^ = 98%), 4 h (SMD 1.74; 95% CI −4.18 to 7.65; *p* = 0.56; *I*^2^ = 99%), 8 h (SMD −2.45; 95% CI −5.34 to 0.44; p = 0.56; *I*^2^ = 99%), or 24 h (SMD −1.37; 95% CI −3.30 to 0.57; *p* = 0.17; *I*^2^ = 96%) (Figure 5A).

### 3.4 Pain scores at rest or during movement

At rest, ESPB did not achieve a statistically significant reduction in pain severity at 2 h (SMD −0.87; 95% CI −2.98 to 1.24; *p* = 0.42; *I*^2^ = 97%) or at 4 h (SMD −0.43; 95% CI −2.31 to 1.46; p = 0.66; *I*^2^ = 97%) post-operatively (Figure 5B). Similarly, pain scores recorded while patients were mobilizing or coughing also revealed no meaningful separation between groups at 2 h (SMD −0.98; 95% CI −3.00 to 1.04; *p* = 0.34; *I*^2^ = 97%) or 4 h (SMD −0.89; 95% CI −2.57 to 0.80; *p* = 0.30; *I*^2^ = 96%) (Figure 5C). However, time to first rescue analgesic was prolonged by ESPB (SMD 4.04; 95% CI 0.77 to 7.31; *p* = 0.02, *I*^2^ = 99%) (Supplementary Figure 2).

### 3.5 Postoperative complications

Two trials (12, 22) reported the incidence of postoperative nausea, and pooled analysis revealed no significant difference between the ESPB and control groups (RR = 1.15; 95% CI, 0.15–8.59; *p* = 0.89; *I*^2^ = 89%). Vomiting data were available from the same pair of studies (12, 22), and no inter-group disparity was detected (RR = 1.01; 95% CI, 0.14–7.32; *p* = 0.99; *I*^2^ = 99%) (Supplementary Figure 1). PONV was documented in two trials (3, 23), pooled results also revealed no meaningful contrast between ESPB and control (RR 0.55; 95% CI 0.23–1.35; *p* = 0.19; *I*^2^ = 36%). Likewise, three studies (3, 12, 22) recorded respiratory depression, with no significant difference observed (RR 1.00; 95% CI 0.40–2.51; *p* = 1.00) (Supplementary Figure 1). Post-operative pruritus was extractable from three reports (12, 22, 23); the aggregated risk was virtually identical in ESPB and control arms (RR 1.14, 95% CI 0.16–8.22; *p* = 0.90; *I*^2^ = 65%), with wide confidence limits reflecting the low event rate (Supplementary Figure 1). Similarly, no case of definite LAST was observed in either group across two investigations that explicitly screened for it (23, 24) (RR 7.00, 95% CI 0.38–130.10; *p* = 0.19).

### 3.6 Other outcome measures

Patient satisfaction with regional analgesia was reported in two trials (23, 24). However, these studies utilized different metrics to assess satisfaction, one analyzing satisfaction scores and the other evaluating the incidence of satisfied patients and neither demonstrated a significant difference between the ESPB and control groups. Our protocol pre-specified evaluation of small-study effects with funnel-plot asymmetry and, if ≥10 trials were available, Egger’s regression and Begg’s rank test. Only six studies contributed to the primary outcome (24-h opioid consumption), so formal tests lack power and were omitted. The funnel plot (Supplementary Figure 3) shows slight asymmetry (one small trial lying outside the pseudo-confidence limits), but with so few data points this could reflect either true heterogeneity or chance. We therefore interpret the pooled estimate cautiously and emphasize the need for additional large RCTs before confident conclusions about ESPB efficacy in arthroscopic shoulder surgery can be drawn.

## 4 Discussion

This systematic review and meta-analysis demonstrated that ESPB lowers the pain scores at 2 and 48 h postoperatively in patients undergoing ASS. Additionally, ESPB also prolonged the first time to rescue analgesic request compared with control group. However, pooled data showed no clinically relevant advantage for ESPB over control in 24-h opioid consumption, or the incidence of PONV, respiratory depression, pruritus or LAST among patients undergoing ASS. Although ESPB may not be superior to ISB (22), it could offer promising postoperative analgesic efficacy compared with no blocks or sham blocks (11, 12). Moreover, CESPB provided alternative and phrenic nerve-sparing analgesic efficacy to ISB (24). ESPB outperformed IAI and PAI and had comparable analgesic efficacy to SNNB (3, 11, 23).

Although opioid sparing and early pain control were the anticipated benefits of ESPB, the demonstrable gains were modest reductions in pain scores at 2 and 48 h and time-to-first rescue analgesia; cumulative morphine use, and all other analgesic metrics remained statistically unchanged. ESPB did not show the merits of increased opioid consumption or the first evidence of analgesic efficacy in patients undergoing ASS. One recent meta-analysis of ESPB in liver surgery involving six studies also revealed that, compared with controls, ESPB did not reduce opioid consumption or lower the pain scores postoperatively (8). This may be a disclosure, though ESPB could offer analgesic efficacy compared with no block or sham block but not be superior to other conventional regional analgesia methods. However, these two meta-analyses only included small sample sizes and insufficient studies with high heterogeneity. Interestingly, ESPB has been employed across a wide spectrum of surgical procedures, reflecting its versatility in selecting the appropriate spinal segment for blockade (5, 7).

A recent investigation demonstrated that, in patients undergoing breast or thoracic procedures, ESPB not only outperformed no-block care but also achieved analgesia comparable to thoracic paravertebral block (TPVB) (5). Moreover, a meta-analysis of 12 trials evaluating ESPB in lumbar surgery found that the block significantly cut 24-h opioid consumption and produced lower pain scores at multiple time-points up to 48 h post-operatively (9). ESPB also increased the patient satisfaction score, decreased the PONV, and minimized the length of hospital stay (9). The inconsistent analgesic profile of ESPB across surgical specialties probably reflects three interacting factors. First, the magnitude of tissue trauma and hence the nociceptive load varies markedly among operations; minimally invasive breast surgery generates a different pain signature from open spinal instrumentation. Second, published RCTs are uniformly underpowered and employ heterogeneous local-anesthetic regimens (ropivacaine 0.2–0.5%, bupivacaine 0.25–0.375%, volumes 15–30 mL), making dose- response inferences impossible. Third, cadaveric and imaging studies (25–27) show that injectate spread and consequent dermatomal anesthesia depend on surgical site: cephalad diffusion is limited after lumbar injection, whereas thoracic deposits reliably reach the paravertebral space and ventral rami, explaining why benefits cluster around thoracic and breast procedures but dissipate in shoulder or hip surgery. Though the majority of studies revealed ESPB, compared with no block or sham block, could offer promising analgesic efficacy by reducing pain scores and opioid consumption, the pain-related results should emphasize the minimally clinically important difference (MCID) (28).

In terms of postoperative complications, despite the theoretical appeal of reducing opioid-related side effects, ESPB failed to translate any putative analgesic gain into higher patient satisfaction or a lower incidence of PONV. This finding is clinically relevant because PONV remains the most distressing opioid-associated complication and a proven driver of prolonged length of stay (29). Indeed, when patients are asked to rank post-operative outcomes, they most wish to avoid, freedom from nausea consistently outranks even pain relief (30). The current evidence therefore indicates that, in the context of ASS, ESPB does not deliver the patient-centered benefit package, such as less PONV, greater satisfaction, which might have been expected from an effective regional technique. One of the included studies showed that a single injection of ESPB was inferior to ISB (22). In contrast to ISB, CESPB may provide an alternative form of postoperative analgesia efficacy (24). ESPB catheterization could extend analgesia and be more effective than a single injection. Furthermore, no serious adverse events, included LAST, pneumothorax, or permanent neurological injury were documented in either the ESPB or control arms. The theoretical safety advantage of ESPB stems from the target plane’s distance from the pleura, major vessels, and neuraxis (31), making catastrophic needle misplacement less likely than with paravertebral or interscalene techniques. Nevertheless, isolated case reports (7) have recorded ropivacaine-induced seizures after high-volume ESPB, and transient ipsilateral motor weakness has been described in several small series (32, 33).

### 4.1 Limitations of current study

The current study is subject to several limitations. First, ESPB is still in the evolutionary stage; investigators have explored different vertebral levels, volumes, concentrations and timings. This legitimate scientific exploration inevitably increases between-study variance and attenuates the strength of any pooled estimate. Second, individual sample sizes were relatively small, the largest ESPB arm contained only 97 patients. Third, high heterogeneity may weekend the reliable of current study due to the heterogenous control arms, variable ESPB protocols, and differences in multimodal analgesia. Fourth, one study published in 2024 we did not include in the analysis due to the search timeline, which may weaken the robustness of the current conclusion (34). Last but not least, the pooled sample is insufficient to confirm the inherent safety of the block; larger surveillance studies are required before rare but potentially severe complications can be confidently quantified.

## 5 Conclusion

This meta-analysis of six RCTs (365 patients) evaluates the analgesic efficacy and safety of ESPB in ASS. ESPB reduces pain scores at 2 and 48 h postoperatively and prolongs time to first rescue analgesic without increasing adverse events (e.g., PONV, respiratory depression). However, it fails to reduce 24-h opioid consumption or early resting/movement pain scores. While ESPB is not superior to ISB, it offers a phrenic nerve-sparing alternative and outperforms intra-articular/peri-articular injections. High heterogeneity and small sample sizes limit conclusions. Standardized ESPB protocols and larger trials are needed to confirm its role in multimodal analgesia for ASS. Certainty of evidence ranged from very low to moderate (GRADE), underscoring the need for larger, methodologically rigorous trials before definitive clinical recommendations can be made.

## Data Availability

The original contributions presented in the study are included in the article/Supplementary material, further inquiries can be directed to the corresponding author.

## Supplementary material

Supplementary Table S1 Sensitivity analysis of postoperative opioid consumption at 24 h (oral morphine equivalent) between ESPB and control group. ESPB, Erector spinae plane block.

Supplementary Figure S1 Forest plot comparing postoperative complications between ESPB and control group.

Supplementary Figure S2 Forest plot comparing the first time of rescue analgesics between ESPB and control group.

Supplementary Figure S3 Funnel plot of the ESPB compared with control groups in the opioid consumption at 24 h postoperatively.
