# Supplementary material for: Analgesic efficacy of erector spinae plane block for managing pain in arthroscopic shoulder surgery: a systemic review and meta-analysis
Source: Front Med (Lausanne). 2025 Dec 12;12:1702898. doi: 10.3389/fmed.2025.1702898 (PMC12746480; doi:10.3389/fmed.2025.1702898)
Supplement: Supplementary Table S1 — Sensitivity analysis of postoperative opioid consumption at 24 h (oral morphine equivalent) between ESPB and control group. ESPB, Erector spinae plane block. [file Table_1.doc]

Supplementary Table1. Sensitivity analysis of postoperative opioid consumption at 24h (oral morphine equivalent) between ESPB and control group. ESPB, Erector spinae plane block.

| Study | Z effect | SMD | 95%CI | I^2^ | P value |
| --- | --- | --- | --- | --- | --- |
| Nagla, et al 2022 | 0.25 | 0.10 | -0.72 to 0.93 | 83% | 0.80 |
| Bahadir, et al 2021 | 1.27 | -1.28 | -3.25 to 0.70 | 97% | 0.20 |
| Lisa, et al 2022 | 1.63 | -1.46 | -3.21 to 0.30 | 97% | 0.10 |
| Furkan, et al 2021 | 1.74 | -1.59 | -3.37 to 0.20 | 97% | 0.08 |
