# Supplementary material for: Analgesic efficacy of erector spinae plane block for managing pain in arthroscopic shoulder surgery: a systemic review and meta-analysis
Source: Front Med (Lausanne). 2025 Dec 12;12:1702898. doi: 10.3389/fmed.2025.1702898 (PMC12746480; doi:10.3389/fmed.2025.1702898)

Supplementary Figure 1: Forest plot comparing postoperative complications between ESPB and control group.


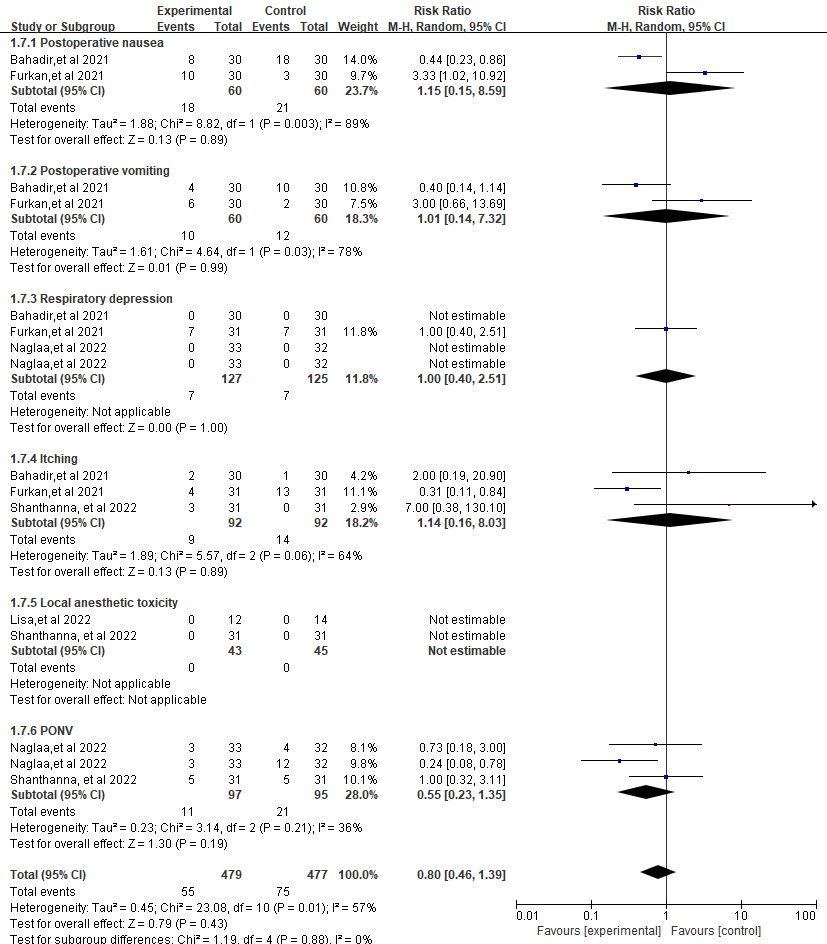


Supplementary Figure 2: Forest plot comparing the first time of rescue analgesics between ESPB and control group.


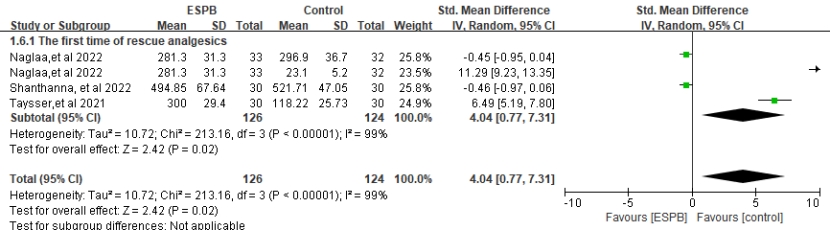


Supplementary Figure 3: Funnel plot of the ESPB compared with control groups in the opioid consumption at 24 h postoperatively.


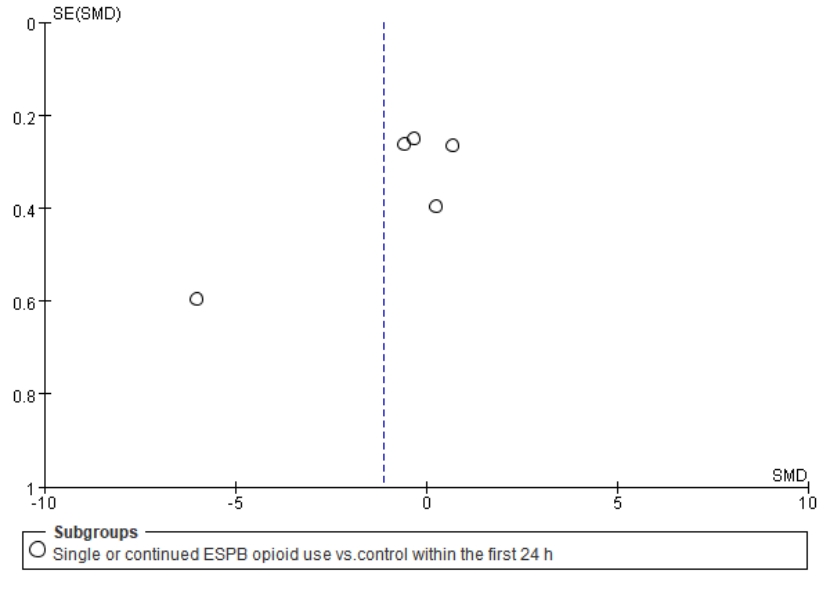

Supplement: Supplementary Figure S1 — Forest plot comparing postoperative complications between ESPB and control group. [file Data_Sheet_2.doc]
