# Supplementary material for: Analgesic efficacy of erector spinae plane block for managing pain in arthroscopic shoulder surgery: a systemic review and meta-analysis
Source: Front Med (Lausanne). 2025 Dec 12;12:1702898. doi: 10.3389/fmed.2025.1702898 (PMC12746480; doi:10.3389/fmed.2025.1702898)
Supplement: Supplementary file 3 [file Data_Sheet_1.doc]

| #1 | **(Erector Spinae Plane Block[Title/Abstract]) OR (Erector Spinae Muscle[Title/Abstract])** |
| --- | --- |
| #2 | **(((Arthroscopic Shoulder Surgery[Title/Abstract]) OR (Shoulder Surgery[Title/Abstract])) OR (total shoulder arthroplasty[Title/Abstract])) OR (shoulder arthroscopy[Title/Abstract])** |
| #3 | #1 AND #2 |

Pubmed: 03/2023, n=13

Web of Science：03/2023, n=23

| #1 | (TS=(Erector Spinae Plane Block)) OR TS=(Erector Spinae Muscle) |
| --- | --- |
| #2 | (((TS=(Arthroscopic Shoulder Surgery)) OR TS=(Shoulder Surgery)) OR TS=(total shoulder arthroplasty)) OR TS=(shoulder arthroscopy) |
| #3 | #1 AND #2 |

The Cochrane library:03/2023,n=36

| #1 | (Erector Spinae Plane Block):ti,ab,kw OR (Erector Spinae Muscle):ti,ab,kw |
| --- | --- |
| #2 | (Arthroscopic Shoulder Surgery):ti,ab,kw OR (Shoulder Surgery):ti,ab,kw OR (total shoulder arthroplasty):ti,ab,kw OR (shoulder arthroscopy):ti,ab,kw |
| #3 | #1 AND #2 |

*Embase: 03/2023, n=27*

| #1 | 'erector spinae muscle' or 'erector spinae block' or 'erector spinae plane block' or 'erector spinae' |
| --- | --- |
| #2 | 'shoulder surgery' or 'arthroscopic shoulder surgery' or 'shoulder arthroscopy' or 'total shoulder arthroplasty' |
| #3 | #1 AND #2 |
